# Exposure to e-cigarette advertising and young people’s use of e-cigarettes: A four-country study

**DOI:** 10.18332/tid/172414

**Published:** 2023-10-24

**Authors:** Simone Pettigrew, Joseph A. Santos, Ana-Catarina Pinho-Gomes, Yuan Li, Alexandra Jones

**Affiliations:** 1 The George Institute for Global Health, University of New South Wales, Sydney, Australia; 2 The George Institute for Global Health, London, United Kingdom; 3 School of Public Health, Imperial College London, London, United Kingdom; 4 Institute of Health Informatics, University College London, London, United Kingdom; 5 The George Institute for Global Health, Beijing, China

**Keywords:** e-cigarettes, vaping, advertising, promotion, marketing

## Abstract

**INTRODUCTION:**

The World Health Organization recommends banning all forms of e-cigarette advertising, promotion, and sponsorship. The aims of the present study were to: 1) examine young people’s exposure to e-cigarette advertising across a wide range of media in four diverse countries; and 2) identify any association between the number of different types of media exposures and e-cigarette use.

**METHODS:**

A cross-sectional online survey was administered to approximately 1000 people aged 15–30 years in Australia, China, India, and the United Kingdom (n=4107). The survey assessed demographic characteristics, e-cigarette and tobacco use, numbers of friends and family members who vape, and exposure to multiple forms of e-cigarette advertising (e.g. television, radio, print, and various types of social media). Descriptive analyses were conducted on those who had heard of e-cigarettes (n=3095, significance threshold p<0.001) and a logistic regression analysis was used to identify factors associated with e-cigarette ever use (significance threshold p<0.05).

**RESULTS:**

The majority (85%) of respondents who had heard of e-cigarettes reported being exposed to e-cigarette advertising on at least one type of media, and the average number of types of media to which respondents were exposed was 5 (range: 0–17). The number of media types was significantly associated with ever use of e-cigarettes (OR=1.05; 95% CI: 1.02–1.08, p=0.001).

**CONCLUSIONS:**

Despite advertising restrictions in place in all four countries, large majorities of young people reported being exposed to e-cigarette advertising. Social media and advertising on/around vape shops and other retailers appear to be key exposure locations. Urgent attention is needed to address these forms of exposure given their apparent association with e-cigarette use.

## INTRODUCTION

The use of e-cigarettes by children, adolescents, and young adults is of substantial public health concern due to the potential harms associated with exposure to nicotine by the developing brain and the heightened risk of addiction resulting from greater brain plasticity in these stages of life^1^. Perceptions of harmfulness and addictiveness have been found to be inversely associated with usage intentions and actual use of e-cigarettes among youth^2-4^. However, exposure to e-cigarette advertising can reduce risk perceptions and stimulate curiosity, increasing susceptibility to use^5-7^. There is some evidence of an inverse relationship between young people’s harm perceptions and the number of different types of media via which they are exposed to e-cigarette advertising^5^.

As part of a suite of strategies to reduce uptake of vaping among minors and non-smokers, the World Health Organization recommends banning all forms of e-cigarette advertising, promotion, and sponsorship^8^. This is consistent with Article 13 of the Framework Convention on Tobacco Control that requires signatories to ban the advertising, promotion, and sponsorship of tobacco products^9^. However, a growing body of evidence demonstrates that regardless of regulatory frameworks in place, young people are routinely exposed to promotional material for e-cigarettes^3,5,10-15^. This is often due to the use of Internet-based advertising, especially social media^6,13,16^. Digital forms of e-cigarette promotion are particularly difficult to monitor and control, and effective regulation can require cross-border arrangements^8^. To inform such efforts, evidence is needed of the specific forms of e-cigarette advertising to which young people are exposed, to ensure appropriate restrictions are put in place.

The aim of the present study was to examine young people’s exposure to e-cigarette advertising across a wide range of media and identify any association between the number of different types of media exposures and e-cigarette use. To contribute to the limited work in this field, much of which has been conducted in the United States^2,7,17-21^, data were collected in four countries: Australia, China, India, and the United Kingdom (UK). These four countries have varying regulatory frameworks relating to the supply and promotion of e-cigarettes. Australia has a prescription-only model for nicotine e-cigarettes used for cessation purposes, but non-nicotine e-cigarettes can be purchased legally in most states. There is a general ban on advertising, although pharmacies are permitted to communicate information about the availability of prescribed e-cigarette products^22^. China has banned non-nicotine e-cigarettes and all e-cigarette advertising, but nicotine e-cigarettes can be sold as regular consumer goods to those aged ≥18 years^23^. India has amongst the strictest regulatory environments in the world: nicotine and non-nicotine e-cigarettes are banned and advertising is not permitted^24^. The UK has the most liberal laws across the four included countries. Both nicotine and non-nicotine e-cigarettes can be legally sold to adults (≥18 years), but advertising is not permitted on television, radio, print media, and the Internet^25^. These diverse national contexts with substantial regulatory and cultural variation provide access to heterogenous environments for the purpose of assessing exposure to e-cigarette advertising despite comprehensive bans and investigating the relationship between exposure to e-cigarette advertising and e-cigarette use.

## METHODS

This project was undertaken as part of a four-country (Australia, China, India, and UK) study examining various factors associated with e-cigarette use among young people^3,10,11,26^. A survey was administered to samples of approximately 1000 people aged 15–30 years in each country. An ISO-accredited international web panel provider (Pureprofile) undertook recruitment and hosted the survey. Quotas were applied to achieve within each country a sample comprising approximately equal numbers of males and females and representation in each year group. During recruitment, a survey link was disseminated to eligible web panel members inviting them to participate in a survey that would take around 15 minutes to complete. No specific topic was specified in the invitation email, and respondents were unaware of the focus on e-cigarettes until entering the survey and reading the study information sheet. Respondents from Australia and the UK were served an English version of the survey instrument, while respondents from China or India could choose to complete the survey in either English, or in Mandarin or Hindi, respectively.

The survey was in field November–December 2021. The survey completion rate among respondents meeting age and quota fulfilment eligibility criteria was 82%. Of these respondents, 8% were removed from the sample following data quality checks that included identification of instances where the survey was completed multiple times from the same device, completion time was too short for appropriate participation, and excessive ‘straight line’ responses were provided (i.e. little to no variation in response options selected). The study received approval from a University Human Research Ethics Committee and respondents provided informed consent.

Assessed demographic attributes included sex, age, income, and education level. Respondents were asked if they had ever heard of e-cigarettes, which were described as follows: ‘Electronic cigarettes or e-cigarettes are personal vaporizing devices where users inhale vapor rather than smoke. E-cigarettes often contain flavors and might contain nicotine. They are also known as e-cigs, vaporizers, vapes, vape pens, vape pods, Juul, mods, HQD Cuvies, Puff Bars, and Stigs’. Those responding in the affirmative were asked whether they ‘Never used’, ‘Previously used’ (‘even just once or twice’), or ‘Currently use’ the products^27^. For consistency, the same items were used to determine smoking status. Respondents who had heard of e-cigarettes were asked how many of their family and friends used e-cigarettes^28^.

Exposure to e-cigarette advertising among respondents who had heard of e-cigarettes was assessed across multiple items. The following items were adapted from the US National Youth Tobacco Survey^29^: ‘When you are using the internet, how often do you see ads or promotions for e-cigarettes when you are not searching for e-cigarettes?’, and ‘When you are on social media, how often do you see posts related to e-cigarettes?’, with responses required for each major social media platform; ‘When you watch TV, streaming services (such as Netflix), or a movie at the cinema, how often do you see ads or promotions for e-cigarettes, or entertainers (actors, influencers etc.) using e-cigarettes?’; and ‘When you go to a convenience store, supermarket, or petrol station, how often do you see advertisements or promotions for e-cigarettes?’. All these items had four response options: 1 (‘Never’) to 4 (‘Often’), with an additional option ‘I do not use (the nominated media)’. An additional item asked: ‘Where else have you seen advertisements, promotions, or other marketing material for e-cigarettes?’, with response options including billboards, magazines, radio, kiosks, tobacconists, vape shops, and bottle shops/liquor stores (Yes/No)^17^. This resulted in respondents being asked to provide exposure information relating to 17 forms of media. Of these, seven represented online media (Internet sites and social media) and the remainder were classified as being ‘in real life’ (e.g. in magazines or at tobacconists).

### Analysis

Descriptive analysis was conducted for each media type, with chi-squared analyses used to identify significant differences in exposure by e-cigarette user status. Due to the large number of comparisons, a significance threshold of p<0.001 was applied. Mixed effects logistic regression analysis was used to assess whether the number of media on which respondents were exposed to e-cigarette advertising was associated with ever (current or previous) e-cigarette use, controlling for demographic, social, and tobacco use variables (significance threshold p<0.05). A random intercept by country was included in the model to take into account the clustering effect by country. An advertising exposure composite score was calculated for each respondent by tallying the number of advertising media on which e-cigarette advertising was seen. Analyses were conducted using Stata BE version 17.

## RESULTS

The sample profile for each country and overall is shown in Supplementary file Table 1. Of the total sample of 4107 respondents, 1011 reported never having heard of e-cigarettes and were excluded from further analyses. Most of the ‘never heard’ respondents were from China (50% of the never-heard group) and India (35%).

Reported exposure to e-cigarette advertising for each media type by e-cigarette user status for the aggregated sample is shown in Table 1, and results for individual countries are reported in Supplementary file Tables 2–5. Across all four countries, 85% of respondents had been exposed to e-cigarette advertising on at least one type of media, ranging from 79% for never users to 95% for current users. The average number of media types to which respondents were exposed was 5 (range: 0–17).

**Table 1:** Descriptive results for e-cigarette advertising exposure by location and e-cigarette use status among surveyed people aged 15–30 years, November–December 2021 (Australia, China, India, and UK samples combined) (N=3095)

*Type of advertisement exposure*	*Total n (%)*	*Never users n (%)*	*Previous users n (%)*	*Current users n (%)*	*Significant differences*
	*(N=3095)*	*(N=1589)*	*(N=914)*	*(N=592)*	
**In real life**					
Vape shops^†^	1474 (48)	587 (37)	514 (56)	373 (63)	a, b
Supermarket/corner store/petrol station^§^	1290 (42)	521 (33)	415 (45)	354 (60)	a, b, c
Tobacconists^†^	853 (28)	350 (22)	311 (34)	192 (32)	a, b
TV, cinema, streaming services^§^	843 (27)	324 (20)	258 (28)	261 (44)	a, b, c
Magazines^†^	708 (23)	337 (21)	208 (23)	163 (28)	NS
Kiosks^†^	648 (21)	285 (18)	213 (23)	150 (25)	b
Bottle shops or liquor stores^†^	574 (19)	263 (17)	173 (19)	138 (23)	b
Billboards^†^	480 (16)	215 (14)	145 (16)	120 (20)	b
Radio^†^	372 (12)	166 (10)	108 (12)	98 (17)	b
**Online**					
Internet	911 (29)	355 (22)	285 (31)	271 (46)	a, b, c
**Social media^‡^**					
**Australia, India, UK**	**(N=2535)**	**(N=1243)**	**(N=787)**	**(N=505)**	
Instagram	997 (39)	390 (31)	313 (40)	294 (58)	a, b, c
YouTube	916 (36)	363 (29)	280 (36)	273 (54)	b, c
Facebook	868 (34)	328 (26)	266 (34)	274 (54)	a, b, c
TikTok	849 (33)	295 (24)	281 (36)	273 (54)	a, b, c
Snapchat	778 (31)	270 (22)	267 (34)	241 (48)	a, b, c
Twitter	565 (22)	199 (16)	166 (21)	200 (40)	b, c
Pinterest	459 (18)	169 (14)	150 (19)	140 (28)	b, c
**China**	**(N=560)**	**(N=346)**	**(N=127)**	**(N=87)**	
Douyin	281 (50)	137 (40)	79 (62)	65 (75)	a, b
WeChat	255 (46)	126 (36)	65 (51)	64 (74)	b
Xiao Hong Shu (Little Red Book)	234 (42)	112 (32)	56 (44)	66 (76)	b, c
Sina Weibo	231 (41)	120 (35)	54 (43)	57 (66)	b
Tencent QQ	213 (38)	108 (31)	51 (40)	54 (62)	b
Zhihu	208 (37)	105 (30)	46 (36)	57 (66)	b, c
Douban	179 (32)	90 (26)	41 (32)	48 (55)	b
**Any type of media**	2645 (85)	1263 (79)	818 (90)	564 (95)	a, b, c

Excludes those who had not heard of e-cigarettes/vaping (n=1011) or had missing data for advertising exposure items (n=1).

† Includes those selecting ‘yes’ from yes/no options.

§ Include ‘Sometimes’ or ‘Often’ (vs ‘Never’ or ‘Rarely’).

‡ Include those selecting ‘Sometimes’ or ‘Often’ (vs ‘Never’, ‘Rarely’, or ‘Don’t use’).

a Never versus previous, significantly different at p<0.001.

b Never versus current, significantly different at p<0.001.

c Previous versus current, significantly different at p<0.001. NS: not significant.

In online contexts, exposure was more common for most social media platforms compared to general Internet usage. For example, 50% of those from China and 39% of those from Australia/India/UK reported seeing e-cigarette advertising on Douyin and Instagram, respectively, compared to 29% seeing e-cigarette advertising when using other parts of the Internet. For ‘in real life’ contexts, exposure was most common for vape shops (48%) and supermarkets/corner stores/petrol stations (42%).

For 23 of 24 media types, those who had never used e-cigarettes were significantly less likely to have been exposed to e-cigarette advertising compared to current users. For half of all media types (12 out of 24), current users were more likely to have been exposed to e-cigarette advertising compared to previous users. For almost half of the media types (10 out of 24), never users were significantly less likely to report exposure compared to previous users.

The results of the logistic regression are shown in Table 2. The number of media types via which respondents had been exposed to e-cigarette advertising was significantly associated with ever e-cigarette use (OR=1.05; 95% CI: 1.02–1.08, p=0.001). This means that the odds of ever cigarette use increased by about 5% per unit increase in e-cigarette advertising exposure. Other significant factors were being a current (OR=16.02; 95% CI: 12.15–21.14, p<0.001) or previous (OR=9.32; 95% CI: 7.32–11.88, p<0.001) tobacco smoker, having friends (OR=4.18; 95% CI: 3.28–5.33, p<0.001) or family members (OR=2.10; 95% CI: 1.66–2.64, p<0.001) who vape, and being male (OR=1.25; 95% CI: 1.02–1.54, p=0.031).

**Table 2:** Logistic regression results: Factors associated with ever e-cigarette use among surveyed people aged 15–30 years, November–December 2021 (Australia, China, India, and UK samples combined) (N=2785*)

*Variables*	*OR*	*95% CI*	*p*
**Age** (years)	0.97	0.95–1.00	0.048
**Sex**			
Female (Ref.)	1		
Male	1.25	1.02–1.54	0.031
Other	1.39	0.40–4.89	0.604
**Tobacco use status**			
Never (Ref.)	1		
Previous	9.32	7.32–11.88	<0.001
Current	16.02	12.15–21.14	<0.001
**Education level**			
School (Ref.)	1		
College certificate or diploma	0.91	0.68–1.22	0.514
University or higher	1.25	0.95–1.65	0.104
**Income**			
Low (Ref.)	1		
Mid	1.18	0.90–1.54	0.227
High	0.93	0.69–1.23	0.598
**Social context^‡^**			
Friends’ e-cig use	4.18	3.28–5.33	<0.001
Family members’ e-cig use	2.10	1.66–2.64	<0.001
Advertising exposure composite score (0–17)^†^	1.05	1.02–1.08	0.001

Country was included in analyses as a random effect. A total of 310 respondents excluded from analyses due to missing data on the education/income variables.

* Excludes those who had not heard of e-cigarettes/vaping (n=1011) or had missing data for advertising exposure items (n=1).

‡ ≥1 members of immediate family/closest friends currently using e-cigarettes.

† The advertising exposure composite score was calculated for each respondent by tallying the number of advertising media on which e-cigarette advertising was seen. e-cig: e-cigarette.

## DISCUSSION

E-cigarette advertising is an important modifiable factor that should be addressed to minimize vaping-related harms^8^. A large majority (85%) of participants in the present study who had heard of e-cigarettes reported being exposed to e-cigarette advertising on at least one type of media. Exposure rates were especially high for vape shops/other retailers and social media, and substantial exposure was also reported across a broad range of other media including television, magazines, billboards, and radio. These results are remarkable, given the regulations in place in all four countries to ban e-cigarette advertising across most, if not all, media.

After controlling for demographic, social, and tobacco use factors identified in previous research as being associated with e-cigarette use status^21,30-32^, the number of media types via which respondents had been exposed to e-cigarette advertising was significantly associated with ever e-cigarette use. This is consistent with previous research demonstrating a link between e-cigarette advertising exposure and susceptibility to/use of e-cigarettes^2,5,17,21,33^, and highlights the importance of ensuring existing advertising regulations are appropriately enforced. Young people in the included countries appear to be in favor of e-cigarette advertising bans^3,10,34^, suggesting that enhanced monitoring and enforcement would likely be supported.

The results of the present study signal the critical importance of restricting e-cigarette advertising on the exterior of vape stores and other retailers, as this appears to be a primary mechanism via which e-cigarette marketers can reach young people. As has been found for alcohol retailing, effective use of signage on and around stores can effectively bypass advertising restrictions to reach vulnerable population groups^35^. Regulations need to be carefully constructed and vigilantly enforced to prevent this exposure.

### Limitations

The primary limitation of this study was the reliance on a web panel provider for respondent recruitment. The strong skew towards higher educated/income respondents in India likely reflects the literacy and technology access requirements of online surveys. Second, the cross-sectional study design prevents causal attributions. It is possible, for example, that e-cigarette users were more attuned to e-cigarette advertising and were therefore more likely to report higher levels of exposure due to greater salience. Third, no attempt was made to distinguish between use or advertising of nicotine and non-nicotine e-cigarettes due to the mis-labelling of these products to evade regulatory restrictions and therefore inability of vapers to be certain of the nature of the products they are using^36^. Fourth, the e-cigarette use measure was quite blunt, and future research could seek to provide a more nuanced account of the nature and extent of young people’s vaping behaviors. Similarly, due to this research being part of a larger international study, the items assessing media exposure were relatively simple in nature. Future work could explore specific media types in more detail using more sensitive exposure measures (e.g. distinguishing between recent and past exposure). Finally, the confined age range and modest number of participating countries limit the generalizability of the results.

## CONCLUSIONS

Despite advertising restrictions in place in all four countries, most young people reported being exposed to e-cigarette advertising. Social media and advertising on/around vape shops and other retailers appear to be key exposure locations. Urgent attention is needed to address these forms of exposure given their apparent association with e-cigarette use.

## Data Availability

The data supporting this research are available from the authors on reasonable request.
