# Supplementary material for: Exposure to e-cigarette advertising and young people’s use of e-cigarettes: A four-country study
Source: Tob Induc Dis. 2023 Oct 24;21:141. doi: 10.18332/tid/172414 (PMC10594952; doi:10.18332/tid/172414)
Supplement: Supplementary file 1 [file TID-21-141-s1.pdf]

Supplementary Table S1: Sample profile for surveyed 15-30 year olds, November-December 2021 (Australia, China, India, and UK samples combined) (n = 4,107)

	**Australia (n = 1006)** n (%)	**China (n = 1062)** n (%)	**India (n = 1030)** n (%)	**UK (n = 1009)** n (%)	**Total (n = 4107)** N (%)
Sex					
Female	511 (51)	553 (52)	508 (49)	520 (52)	2092 (51)
Male	489 (49)	509 (48)	522 (51)	470 (47)	1990 (48)
Other	6 (1)	0 (0)	0 (0)	19 (2)	25 (1)
Age (years)					
15-21	415 (41)	552 (52)	343 (33)	438 (43)	1748 (43)
22-30	591 (59)	510 (48)	687 (67)	571 (57)	2359 (57)
Smoking status					
Never	525 (52)	612 (58)	721 (70)	431 (43)	2289 (56)
Previous	317 (32)	166 (16)	169 (16)	291 (29)	943 (23)
Current	164 (16)	284 (27)	140 (14)	287 (28)	875 (21)
Education					
School	476 (47)	431 (41)	185 (18)	486 (48)	1578 (39)
College certificate/ diploma	184 (18)	385 (36)	131 (13)	182 (18)	882 (22)
University	345 (34)	244 (23)	709 (69)	339 (34)	1637 (40)
Income					
Low	411 (47)	551 (53)	206 (22)	303 (35)	1471 (39)
Mid	350 (40)	342 (33)	106 (11)	302 (35)	1100 (29)
High	111 (13)	155 (15)	640 (67)	262 (30)	1168 (31)
E-cigarette status*	**(n = 930)**	**(n = 560)**	**(n = 676)**	**(n = 930)**	**(n = 3096)**
Never	454 (49)	346 (62)	456 (67)	333 (36)	1589 (51)
Previous	334 (36)	127 (23)	135 (20)	319 (34)	915 (30)
Current	142 (15)	87 (16)	85 (13)	278 (30)	592 (19)

*Excludes those reporting never having heard of e-cigarettes or vaping (n = 1011)

Supplementary Table S2: Descriptive results for e-cigarette advertising exposure by e-cigarette use status among surveyed 15-30 year olds in Australia, November-December 2021 (n=929)

	Total sample n = 929	Never users n = 454	Previous users n = 333	Current users n = 142	Significant differences
**IN REAL LIFE**					
Vape shops~	419 (45%)	179 (39%)	156 (47%)	84 (59%)	b
Supermarket/corner store/petrol station#	218 (23%)	88 (19%)	76 (23%)	54 (38%)	b
Tobacconists~	329 (35%)	133 (29%)	141 (42%)	55 (39%)	a
TV, cinema, streaming services#	154 (17%)	56 (12%)	61 (18%)	37 (26%)	b
Magazines~	122 (13%)	54 (12%)	43 (13%)	25 (18%)	NS
Kiosks~	126 (14%)	54 (12%)	53 (16%)	19 (13%)	NS
Bottle shops or liquor stores~	114 (12%)	48 (11%)	47 (14%)	19 (13%)	NS
Billboards~	55 (6%)	22 (5%)	18 (5%)	15 (11%)	NS
Radio~	67 (7%)	27 (6%)	26 (8%)	14 (10%)	NS
**ONLINE**					
Internet	153 (16%)	56 (12%)	55 (17%)	42 (30%)	b
Social media^					
Instagram	285 (31%)	108 (24%)	101 (30%)	76 (54%)	b,c
YouTube	202 (22%)	70 (15%)	70 (21%)	62 (44%)	b,c
Facebook	223 (24%)	74 (16%)	81 (24%)	68 (48%)	b,c
TikTok	287 (31%)	102 (22%)	107 (32%)	78 (55%)	b,c
Snapchat	236 (25%)	77 (17%)	99 (30%)	60 (42%)	a,b
Twitter	117 (13%)	32 (7%)	41 (12%)	44 (31%)	b,c
Pinterest	92 (10%)	26 (6%)	39 (12%)	27 (19%)	b
**ANY TYPE OF MEDIA**	694 (75%)	297 (65%)	268 (80%)	129 (91%)	a,b

Notes: Excludes those who had not heard of e-cigarettes/vaping

n/% include ‘Sometimes’ or ‘Often’ (vs ‘Never’ or ‘Rarely’); ^ n/% include those selecting ‘Sometimes’ or ‘Often’ (vs ‘Never’, ‘Rarely’, or ‘Don’t use’); ~ n/% includes those selecting ‘yes’ from yes/no options

a = never versus previous significantly different at $p < 0.001$; **b** = never versus current significantly different at $p<0.001$; **c** = previous versus current significantly different at $p < 0.001$

Supplementary Table S3: Descriptive results for e-cigarette advertising exposure by e-cigarette use status among surveyed 15-30 year olds in China, November-December 2021 (n=560)

	Total sample n = 560	Never users n = 346	Previous users n = 127	Current users n = 87	Significant differences
**IN REAL LIFE**					
Vape shops~	295 (53%)	139 (40%)	87 (69%)	69 (79%)	a,b
Supermarket/corner store/petrol station#	248 (44%)	117 (34%)	70 (55%)	61 (70%)	a,b
Tobacconists~	125 (22%)	61 (18%)	35 (28%)	29 (33%)	NS
TV, cinema, streaming services#	179 (32%)	72 (21%)	48 (38%)	59 (68%)	a,b,c
Magazines~	129 (23%)	73 (21%)	32 (25%)	24 (28%)	NS
Kiosks~	185 (33%)	96 (28%)	53 (42%)	36 (41%)	NS
Bottle shops or liquor stores~	95 (17%)	57 (16%)	16 (13%)	22 (25%)	NS
Billboards~	152 (27%)	76 (22%)	45 (35%)	31 (36%)	NS
Radio~	87 (16%)	48 (14%)	16 (13%)	23 (26%)	NS
**ONLINE**					
Internet	218 (39%)	94 (27%)	65 (51%)	59 (68%)	a,b
Social media^					
Douyin	281 (50%)	137 (40%)	79 (62%)	65 (75%)	a,b
WeChat	255 (46%)	126 (36%)	65 (51%)	64 (74%)	b
Xiao Hong Shu (Little Red Book)	234 (42%)	112 (32%)	56 (44%)	66 (76%)	b,c
Sina Weibo	231 (41%)	120 (35%)	54 (43%)	57 (66%)	b
Tencent QQ	213 (38%)	108 (31%)	51 (40%)	54 (62%)	b
Zhihu	208 (37%)	105 (30%)	46 (36%)	57 (66%)	b,c
Douban	179 (32%)	90 (26%)	41 (32%)	48 (55%)	b
**ANY TYPE OF MEDIA**	531 (95%)	318 (92%)	126 (99%)	87 (100%)	NS

Notes: Excludes those who had not heard of e-cigarettes/vaping
n/% include 'Sometimes' or 'Often' (vs 'Never' or 'Rarely'); ^ n/% include those selecting 'Sometimes' or 'Often' (vs 'Never', 'Rarely', or 'Don't use'); ~ n/% includes those selecting 'yes' from yes/no options
a = never versus previous significantly different at $p < 0.001$; **b** = never versus current significantly different at $p<0.001$; **c** = previous versus current significantly different at $p < 0.001$

Supplementary Table S4: Descriptive results for e-cigarette advertising exposure by e-cigarette use status among surveyed 15-30 year olds in India, November-December 2021 (n = 676)

	Total sample n = 676	Never users n = 456	Previous users n = 135	Current users n = 85	Significant differences
**IN REAL LIFE**					
Vape shops~	208 (31%)	94 (21%)	66 (49%)	48 (56%)	a,b
Supermarket/corner store/petrol station#	252 (37%)	133 (29%)	66 (49%)	53 (62%)	a,b
Tobacconists~	163 (24%)	82 (18%)	46 (34%)	35 (41%)	a,b
TV, cinema, streaming services#	254 (38%)	144 (32%)	56 (41%)	54 (64%)	b
Magazines~	251 (37%)	148 (32%)	59 (44%)	44 (52%)	NS
Kiosks~	96 (14%)	47 (10%)	23 (17%)	26 (31%)	b
Bottle shops or liquor stores~	161 (24%)	88 (19%)	40 (30%)	33 (39%)	b
Billboards~	105 (16%)	51 (11%)	28 (21%)	26 (31%)	b
Radio~	101 (15%)	58 (13%)	20 (15%)	23 (27%)	NS
**ONLINE**					
Internet	205 (30%)	104 (23%)	55 (41%)	46 (54%)	a,b
Social media^					
Instagram	319 (47%)	181 (40%)	81 (60%)	57 (67%)	a,b
YouTube	324 (48%)	189 (41%)	82 (61%)	53 (62%)	a,b
Facebook	299 (44%)	162 (36%)	79 (59%)	58 (68%)	a,b
TikTok	178 (26%)	99 (22%)	43 (32%)	36 (42%)	b
Snapchat	213 (32%)	115 (25%)	51 (38%)	47 (55%)	b
Twitter	192 (28%)	104 (23%)	49 (36%)	39 (46%)	b
Pinterest	189 (28%)	101 (22%)	51 (38%)	37 (44%)	a,b
**ANY TYPE OF MEDIA**	566 (84%)	362 (79%)	121 (90%)	83 (98%)	b

Notes: Excludes those who had not heard of e-cigarettes/vaping

n/% include ‘Sometimes’ or ‘Often’ (vs ‘Never’ or ‘Rarely’); ^ n/% include those selecting ‘Sometimes’ or ‘Often’ (vs ‘Never’, ‘Rarely’, or ‘Don’t use’); ~ n/% includes those selecting ‘yes’ from yes/no options

a = never versus previous significantly different at $p < 0.001$; **b** = never versus current significantly different at $p<0.001$; **c** = previous versus current significantly different at $p < 0.001$

Supplementary Table S5: Descriptive results for e-cigarette advertising exposure by e-cigarette use status among surveyed 15-30 year olds in the UK, November-December 2021 (n = 930)

	Total sample n = 930	Never users n = 333	Previous users n = 319	Current users n = 278	Significant differences
**IN REAL LIFE**					
Vape shops~	552 (59%)	175 (53%)	205 (64%)	172 (62%)	NS
Supermarket/corner store/petrol station#	572 (62%)	183 (55%)	203 (64%)	186 (67%)	NS
Tobacconists~	236 (25%)	74 (22%)	89 (28%)	73 (26%)	NS
TV, cinema, streaming services#	256 (28%)	52 (16%)	93 (29%)	111 (40%)	a,b
Magazines~	206 (22%)	62 (19%)	74 (23%)	70 (25%)	NS
Kiosks~	241 (26%)	88 (26%)	84 (26%)	69 (25%)	NS
Bottle shops or liquor stores~	204 (22%)	70 (21%)	70 (22%)	64 (23%)	NS
Billboards~	168 (18%)	66 (20%)	54 (17%)	48 (17%)	NS
Radio~	117 (13%)	33 (10%)	46 (14%)	38 (14%)	NS
**ONLINE**					
Internet	335 (36%)	101 (30%)	110 (34%)	124 (45%)	b
Social media^					
Instagram	393 (42%)	101 (30%)	131 (41%)	161 (58%)	b,c
YouTube	390 (42%)	104 (31%)	128 (40%)	158 (57%)	b,c
Facebook	346 (37%)	92 (28%)	106 (33%)	148 (53%)	b,c
TikTok	384 (41%)	94 (28%)	131 (41%)	159 (57%)	b,c
Snapchat	329 (35%)	78 (23%)	117 (37%)	134 (48%)	a,b
Twitter	256 (28%)	63 (19%)	76 (24%)	117 (42%)	b,c
Pinterest	178 (19%)	42 (13%)	60 (19%)	76 (27%)	b
**ANY TYPE OF MEDIA**	854 (92%)	286 (86%)	303 (95%)	265 (95%)	a,b

Notes: Excludes those who had not heard of e-cigarettes/vaping
n/% include 'Sometimes' or 'Often' (vs 'Never' or 'Rarely'); ^ n/% include those selecting 'Sometimes' or 'Often' (vs 'Never', 'Rarely', or 'Don't use'); ~ n/% includes those selecting 'yes' from yes/no options
a = never versus previous significantly different at $p < 0.001$; **b** = never versus current significantly different at $p<0.001$; **c** = previous versus current significantly different at $p < 0.001$

© 2023 Pettigrew S. et al.
